# The neural basis of theory of mind and its relationship to social functioning and social anhedonia in individuals with schizophrenia^☆^

**DOI:** 10.1016/j.nicl.2013.11.006

**Published:** 2013-11-27

**Authors:** David Dodell-Feder, Laura M. Tully, Sarah Hope Lincoln, Christine I. Hooker

**Affiliations:** Department of Psychology, Harvard University, Cambridge, MA 02138, USA

**Keywords:** Schizophrenia, Theory of mind, Social functioning, Social anhedonia, fMRI

## Abstract

Theory of mind (ToM), the ability to attribute and reason about the mental states of others, is a strong determinant of social functioning among individuals with schizophrenia. Identifying the neural bases of ToM and their relationship to social functioning may elucidate functionally relevant neurobiological targets for intervention. ToM ability may additionally account for other social phenomena that affect social functioning, such as social anhedonia (SocAnh). Given recent research in schizophrenia demonstrating improved neural functioning in response to increased use of cognitive skills, it is possible that SocAnh, which decreases one's opportunity to engage in ToM, could compromise social functioning through its deleterious effect on ToM-related neural circuitry. Here, twenty individuals with schizophrenia and 18 healthy controls underwent fMRI while performing the False-Belief Task. Aspects of social functioning were assessed using multiple methods including self-report (Interpersonal Reactivity Index, Social Adjustment Scale), clinician-ratings (Global Functioning Social Scale), and performance-based tasks (MSCEIT—Managing Emotions). SocAnh was measured with the Revised Social Anhedonia Scale. Region-of-interest and whole-brain analyses revealed reduced recruitment of medial prefrontal cortex (MPFC) for ToM in individuals with schizophrenia. Across all participants, activity in this region correlated with most social variables. Mediation analysis revealed that neural activity for ToM in MPFC accounted for the relationship between SocAnh and social functioning. These findings demonstrate that reduced recruitment of MPFC for ToM is an important neurobiological determinant of social functioning. Furthermore, SocAhn may affect social functioning through its impact on ToM-related neural circuitry. Together, these findings suggest ToM ability as an important locus for intervention.

## Highlights

• Individuals with schizophrenia exhibited reduced recruitment of MPFC for ToM.
• MPFC and RTPJ activities correlate with measures of social functioning and ability.
• MPFC activity mediates the relationship between social anhedonia and functioning.
• Neural circuitry supporting ToM may represent an important area for remediation.

## 1 Introduction

Social functioning impairment is a hallmark of schizophrenia that is not amenable to most common forms of treatment (Swartz et al., 2007). Elucidating determinants of social functioning are thus a critical step in the development of effective interventions. Research in this area however, may be limited by a lack of information regarding the neural mechanisms that give rise to social impairment. Such information may elucidate the neurocognitive processes and neural substrates to be targeted for remediation that will most likely result in functional improvements.

Theory of mind (ToM), or the ability to attribute and reason about the mental states of others, is markedly impaired in individuals with schizophrenia (Bora et al., 2009; Sprong et al., 2007). Critically, among individuals with schizophrenia, these deficits have been consistently linked to aspects of social functioning (Couture et al., 2006; Fett et al., 2011; Horan et al., 2012), are more proximal to daily functioning (Bora et al., 2006; Couture et al., 2011; McGlade et al., 2008), and account for more of the variance in daily functioning than other aspects of the illness, including non-social aspects of cognition and symptoms (Brune et al., 2007; Fett et al., 2011; Kosmidis et al., 2011; Pinkham and Penn, 2006). Though the neural bases of ToM have been fairly well characterized in healthy adults, encompassing right and left temporo-parietal junctions (RTPJ, LTPJ) and medial prefrontal cortex (MPFC) (Mar, 2011; Van Overwalle, 2009), less is known about the ToM network in individuals with schizophrenia. The extant literature suggests functional and anatomical abnormalities in MPFC (Benedetti et al., 2009; Brune et al., 2011, 2003; Das et al., 2012; Hooker et al., 2011; Lee et al., 2006, 2011; Walter et al., 2009a; Yamada et al., 2007). However, findings are inconsistent, and, surprisingly, few such studies investigate the relationship between neural activity for ToM and social functioning. Such data could provide biomarkers of social dysfunction, which may prospectively predict schizophrenia outcomes, and neural targets for remediation. Thus, the first aim of the current study was to characterize the neural bases of ToM in schizophrenia and investigate their relationship to aspects of social behavior using multiple methods of assessment.

Social anhedonia, characterized by a trait-like disinterest and lack of pleasure from social interaction, is another important determinant of social functioning in individuals with schizophrenia (Blanchard et al., 1998; Cohen et al., 2005) and the general population (Blanchard et al., 2011). Individuals with high levels of social anhedonia, which is an enduring feature of schizophrenia-spectrum disorders (Blanchard et al., 1998, 2001; Horan et al., 2008), are more likely to be socially isolated (Brown et al., 2007; Kwapil et al., 2009), report less social support and social coping (Blanchard et al., 2011; Horan et al., 2007), greater levels of perceived stress (Horan et al., 2007), and worse functioning within the family unit including less family cohesion, support, and more conflict (Blanchard et al., 2011). Much of the extant literature concerning the underlying mechanisms connecting social anhedonia to these aforementioned consequences for social functioning has focused on the role of diminished anticipatory reward for future social interaction (Barch and Dowd, 2010; Gard et al., 2007; Horan et al., 2008; Kring and Elis, 2012), which may be a consequence of difficulty representing reward value and subsequently accessing these representations due to impairments in working and episodic memory (Gold et al., 2008; Strauss and Gold, 2012). These findings have led to suggestions that targeting reward-related neural circuitry, possibly through pharmacological means, may ameliorate symptoms of anhedonia and subsequently improve functional outcome (Juckel et al., 2006a, 2006b; Schlagenhauf et al., 2008; Walter et al., 2009b; Waltz et al., 2007).

An alternative uninvestigated possibility that would carry important treatment implications is that social anhedonia impacts social functioning, at least partially, through its consequences on neural circuitry supporting social cognitive processes, such as ToM. Substantial evidence exists that repeated engagement in cognitive skills, as in cognitive remediation treatments for schizophrenia, improves neural function due to the brain's capacity for reorganization in response to environmental input (Buonomano and Merzenich, 1998; Eack et al., 2010; Hooker et al., 2012; Penades et al., 2013; Subramaniam et al., 2012). Likewise, both animal and human studies have demonstrated that failure to engage in cognitive skills, via social deprivation, for example, can produce profound neurobiological alterations (Barr et al., 2004; Chugani et al., 2001; Geyer et al., 1993; Kaufman et al., 2000; Suomi, 1997). Considering these findings in the context of social anhedonia, social isolation may remove opportunities to engage in ToM and this lack of engagement may precipitate a cascade of aberrant neuroplastic events that result in disruption to neural networks supporting social cognition (Hoffman, 2007). These disrupted neural networks may, in turn, manifest as difficulty in inferring the intentions and emotions of others, which then contributes to the interpersonal difficulties, compromised social networks, and further isolation that characterizes social functioning impairment in schizophrenia and individuals with high levels of social anhedonia.

If disruption to ToM-related neural circuitry accounts for the relationship between social anhedonia and social functioning, it would suggest ToM as a more proximal process to social functioning than social anhedonia. This has significant implications for schizophrenia as it means that neurocognitive improvements in ToM may more directly support improved social functioning. In other words, it would suggest ToM as a more effective treatment target than social anhedonia or anhedonia more broadly. Furthermore, the early presence of social anhedonia and accompanying withdrawal pre-illness onset (Gooding et al., 2005; Kwapil, 1998; Tarbox and Pogue-Geile, 2008), and its temporal stability (Blanchard et al., 1998, 2001), suggest that social anhedonia could be a marker of risk for disruption to ToM-related neural circuitry and subsequent social dysfunction. Thus, engaging and training ToM processes early in development in those exhibiting social withdrawal may help to prevent social dysfunction (Cornblatt et al., 2012; Tarbox and Pogue-Geile, 2008). In consideration of these issues, our second aim was to investigate this proposed relationship; that is, whether neural activity for ToM accounts for the link between social anhedonia and social functioning.

We addressed these aims using a well-validated ToM scanner task ubiquitously employed in the social neuroscience literature: the False-Belief Task (Saxe and Kanwisher, 2003). Between-group differences were examined in a priori regions-of-interest (ROIs) identified from the ToM literature and with whole-brain analyses. The use of ROIs increases the certainty that the neural activity under examination relates to the process of mental state attribution as opposed to illness-related deficits in peripheral cognitive processes (Poldrack, 2006; Saxe et al., 2006). Furthermore, we investigate how neural activity for ToM relates to different aspects of social behavior (i.e., trait empathy/perspective taking, the ability to manage emotions in different social contexts, the quantity/quality of social interaction in a variety of social roles) with multiple methods of assessment (i.e., self-report, clinician-ratings, performance-based measures). Converging evidence from these assessments make it less likely for significant relationships to be an artifact of the method used (Hooker et al., 2011). Mediation models were used to test whether neural activity for ToM accounts for the relationship between social anhedonia and social functioning. We predict the following: 1) individuals with schizophrenia will exhibit reduced recruitment of the ToM network, specifically MPFC, compared to matched controls; 2) neural activity in these regions will predict social behavior across all participants; 3) neural activity in these regions will mediate the relationship between social anhedonia and social functioning.

## 2 Methods

### 2.1 Participants

Twenty individuals with schizophrenia or schizoaffective disorder (SZ) and 18 healthy controls (HC) were recruited from the Greater Boston Area and participated for monetary compensation (Table 1). Inclusion criteria for all participants included being between the ages of 18 and 65, English speaking, no neurological or major medical illness, no history of head trauma, no substance abuse within six months, and no current or past history of substance dependence. Inclusion criteria for SZ included a diagnosis of schizophrenia or schizoaffective disorder, no comorbid axis I disorders, and no history of electroconvulsive therapy. Inclusion criteria for HC included no current or past axis I disorder and no first-degree relative with a psychotic disorder. Efforts were made to recruit an HC group that matched the SZ group in demographics and education. Thus, advertisements for HCs specified that we were particularly interested in participants who finished high school, but did not necessarily attend or complete college. Participants gave informed written consent in accordance with the Institutional Review Board at Harvard University.

All participants were administered the Structured Clinical Interview for DSM-IV Disorders (First et al., 2002) to screen for past and current axis I diagnoses, the Wechsler Abbreviated Scale of Intelligence (Wechsler, 1999) to assess IQ, and the MATRICS Cognitive Consensus Battery (Nuechterlein et al., 2008), which assesses several neurocognitive domains including speed of processing, attention/vigilance, verbal learning, visual learning, reasoning and problem solving, and social cognition (see Section 2.3.2). Symptoms in the SZ group were assessed with the Positive and Negative Syndrome Scale (Kay et al., 1987). Clinical assessments (including the assessments described in Section 2.3) were conducted by MA-level doctoral students in clinical psychology (LMT, SHL) supervised by a licensed clinical psychologist (CIH).

### 2.2 Social anhedonia

Social anhedonia was assessed with the Revised Scale for Social Anhedonia (SocAnh) (Eckblad et al., 1982), which is widely used in the schizophrenia/schizotypy literature to assess this phenomenon (Gooding et al., 2005; Horan et al., 2008; Kwapil, 1998). SocAnh consists of 40 self-reported items answered true/false (e.g., “Having close friends is not as important to me as many people say” [keyed true]; “If given the choice, I would much rather be with others than alone” [keyed false]). Higher scores denote greater disinterest/less pleasure in and lack of social interaction.

### 2.3 Social variables

#### 2.3.1 Interpersonal Reactivity Index

The Interpersonal Reactivity Index (IRI) (Davis, 1980, 1983) is a 28-item self-report measure that assesses different facets of empathy. We were interested in two subscales: the perspective-taking subscale (IRI-PT), which assesses an individual's tendency to engage in ToM, specifically through adopting another's perspective (e.g., “I sometimes try to understand my friends better by imagining how things look from their perspective.”), and the empathic concern subscale (IRI-EC), which assesses an individual's tendency to consider the emotional states and feel sympathy for others (e.g., “I often have tender, concerned feelings for people less fortunate than me.”). Each subscale consists of 7 items that are rated on a scale from 0 (*does not describe me well*) to 4 (*describes me very well*).

#### 2.3.2 MSCEIT—Managing Emotions

Participants completed the Managing Emotions subtest of the Mayer–Salovey–Caruso Emotional Intelligence Test (MSCEIT-ME) (Mayer et al., 2003), a performance-based assessment of social cognition that measures an individual's understanding of how emotions affect behavior and how to best manage emotions in a variety of social contexts. Participants read short vignettes about story characters and judge how socially effective different actions would be for the story character in managing their emotions. This task was completed as part of the MATRICS battery.

#### 2.3.3 Social Adjustment Scale—Self-Report

The Social Adjustment Scale—Self-Report (SAS) (Weissman et al., 1978) is a 54-item questionnaire that assesses functioning over the past two weeks in six areas: work, social and leisure activities, relationship with extended family, role as a marital partner, parental role, and role within the family unit. Participants answer questions on a 5-point scale that are designed to assess performance, friction with others, interpersonal relationships, and satisfaction within each area. Raw scores were averaged across areas and converted to a gender-adjusted *T* score which represents overall social functioning. Lower scores represent better social functioning.

#### 2.3.4 Global Functioning Social Scale

Participants were administered the clinician-rated Global Functioning Social Scale (GFS) (Cornblatt et al., 2007), which assess the quantity and quality of peer relationships on a scale from 1 (*extreme dysfunction*) to 10 (*superior functioning*).

### 2.4 fMRI task: False-Belief Task

Participants underwent functional magnetic resonance imaging while performing an optimized version of the False-Belief Task (Dodell-Feder et al., 2011). This task, as in other ToM tasks used in the literature (Happe, 1994; Stone et al., 1998), requires participants to predict behavior based on mental states. More specifically, participants read short stories designed to fit one of two conditions: (1) False-Belief (FB) stories describe a protagonist's outdated (i.e., “false”) belief, and actions based on that outdated belief (e.g., “The morning of the high school dance, Barbara placed her high heel shoes under her dress and then went shopping. That afternoon, her sister borrowed the shoes and later put them under Barbara's bed.”), and (2) False-Photograph (FP) stories describe outdated physical states in the world through photographs and maps (e.g., “Old maps of the islands near Titan are displayed in the Maritime museum. Erosion has since taken its toll, leaving only the three largest islands.”). Both stories require the representation of false content. The critical difference is that the false content pertains to beliefs in FB stories and physical states in FP stories. Following the presentation of each story, participants responded to a true/false question (half of which referred to the false representation and half referred to reality) with a button press (e.g., FB: “Barbara gets ready assuming her shoes are under the dress”; FP: “Near Titan today, there are many islands”).

In the scanner, participants saw 10 stories per condition, divided into two functional runs (5 stories presented from each condition in each run). Stories were visually presented for 11 s, followed by the true/false question for 6 s, and finally 12 s of fixation on a center cross. Each functional run lasted 5 min and 2 s. Stories were presented according to two predetermined orders (divided evenly between participants in each group), in which story order was pseudorandomized within and across runs. Stimuli were presented in white font on a black background with Matlab 7.6 using Pyschophysics Toolbox extensions (Brainard, 1997; Kleiner et al., 2007). Accuracy and reaction time (RT) data were collected.

### 2.5 fMRI data acquisition and analysis

fMRI data were collected on a 3T Siemens scanner at Harvard University with echo-planar images (47 sagittal slices, 3 × 3 × 3 mm voxels, TE = 30 ms, TR = 2560 ms, flip angle = 85°). A high-resolution T1-weighted anatomical image was acquired with an MPRAGE sequence (176 sagittal slices, 1 × 1 × 1 mm voxels). Data were analyzed with SPM8 (http://www.fil.ion.ucl.ac.uk/spm/software/spm8/) within the general linear model (GLM) framework. Preprocessing occurred in the following steps: realignment to the mean functional image, co-registration to the anatomical image, normalization to MNI template space, and smoothing with an 8 mm Gaussian kernel. Data were high pass filtered at 128 s. Within each subject, hemodynamic response to each condition was estimated at the start of each story for the duration of the story and true/false judgment. Scans that were ± 3 *SD* from the mean global signal intensity or exceeded 3 mm in movement from the previous volume (identified with the Artifact Detection Tool, http://www.nitrc.org/projects/artifact_detect/) were entered as nuisance regressors to reduce noise.

#### 2.5.1 Neural activity for FB > FP within each group

To verify the expected task-related activity, we first identified neural activity for FB > FP separately within HC and SZ with one-sample *t*-tests. The statistical threshold was set to *p* < .001, *k* > 10, uncorrected for multiple comparisons. Regions that survive correction for multiple comparisons at a voxel-wise *p* < .001, corrected at the cluster-level to *p* < .05, are marked with an asterisk (*) in Table 2.

#### 2.5.2 Between-group differences: ROI analysis

Our main analyses focused on neural activity for FB versus FP within a priori regions, defined independently from the current data, that have been demonstrated in previous studies (Dufour et al., 2013; Mar, 2011; Saxe and Kanwisher, 2003; Saxe and Powell, 2006; Van Overwalle, 2009) to be reliably recruited for ToM: RTPJ, LTPJ, DMPFC, middle MPFC (MMPFC) and VMPFC. These ROIs were defined as 9 mm spheres around the peak coordinates identified from an independent group of 62 healthy adult participants scanned on a similar version of the False-Belief Task reported by Dodell-Feder et al. (2011). There existed substantial overlap between the DMPFC and MMPFC ROIs, and neural activity in these regions for FB > FP was highly correlated, *r* = .89, *p* < .001. Given this spatial and functional similarity, we combined the data from these two ROIs so that each participant had a single value representing neural activity in MPFC.

Contrast estimates, averaging across all voxels in an ROI, were extracted from individual participants for FB > baseline and FP > baseline from the ROIs, and a difference score (FB − FP) was calculated from these data. These values were compared between groups with independent samples *t*-tests using a statistical threshold of *p* < .05 (two-tailed).

#### 2.5.3 Between-group differences: Whole-brain ANOVA

We followed-up the ROI analysis with whole-brain random effects analysis to investigate whether other regions were disrupted during mental state reasoning in schizophrenia. Between-group differences were evaluated with full factorial ANOVA models with group as the between-subjects factor and condition as the within-subjects factor. In this model, contrast images representing the difference between condition and baseline activity (i.e., FB > baseline and FP > baseline) were used. This analysis yielded group ∗ condition interaction effects for HC > SZ and SZ > HC. The statistical threshold was set to *p* < .001, *k* > 10/270 mm to achieve a balance between Type I and Type II error rates—a strategy that is recommended for new fields of study (Lieberman and Cunningham, 2009). Regions that survive correction for multiple comparisons at a voxel-wise *p* < .001, corrected at the cluster-level to *p* < .05, are marked with an asterisk (*) in Table 2.

Given that the SZ group we recruited was older than the HC group, we repeated all analyses controlling for age and report them in Supplementary Tables 1 and 2. We did not control for IQ in these analyses given the shared variance between IQ and group membership (Miller and Chapman, 2001).

### 2.6 Correlations between neural activity for ToM and social variables

First, we tested the zero-order relationships between ROI activity and each of the social variables with Pearson correlations. Second, we conducted partial correlations controlling for the effect of age as well as IQ to evaluate the effect of general intelligence on these relationships (Brune et al., 2007). We expected neural activity for ToM to be related to social behavior regardless of diagnosis so all correlations were conducted across all participants. Follow-up analyses examined correlations within each group. In order to reduce possible bias in the correlations, we used data extracted from the independent ROIs as the measure of neural activity, which did not guarantee neural differences between the groups. The statistical threshold was set to *p* < .05 (two-tailed).

### 2.7 Mediation analysis

Finally, we performed a mediation analysis to investigate the hypothesis that neural activity for ToM accounts for the link between social anhedonia and our measures of functioning (SAS, GFS). Several paths between the variables are estimated in typical mediation analyses including the *total effect* of an independent variable X on a dependent variable Y (path *c*), which consists of the *direct effect* of X on Y after controlling for mediator M (path *c*′) and the *indirect effect* of X on Y through M (i.e., the product of path X ➔ M and M ➔ Y; path *ab*) (Fig. 3A). A test of mediation is conducted by evaluating whether path *ab* is significantly different from zero; that is, whether there exists a significant difference between the *total effect* (path *c*) and *direct effect* (path *c*′) that accounts for M (Preacher and Hayes, 2004, 2008). We used a non-parametric bootstrapping procedure, which is better suited for smaller sample sizes, to derive bias-corrected 95% CIs of the *ab* sampling distribution based on 5000 bootstrap samples (Preacher and Hayes, 2008). If the CI does not encompass zero, then the *indirect effect* (path *ab*) is significantly different from zero (*p* < .05), indicating that neural activity accounts for a statistically significant portion of the relationship between social anhedonia and social functioning. Contrast values extracted from the independent ROIs were used as the measure of neural activity. Data from all participants were used in this analysis.

## 3 Results

### 3.1 Participant characteristics and False-Belief Task performance

SZ and HC did not differ in demographic characteristics or IQ (Table 1). SZ performed worse on all neurocognitive domains assessed in the MATRICS, except working memory, with effect sizes ranges from .36 (visual learning) to .85 (speed of processing). However, only the differences in speed of processing, and verbal learning (at a trend level) were statistically significant. Notably, the difference between SZ and HC on SocAnh was significant only at a trend level and smaller than what has been observed by other investigations (Horan et al., 2008). Behavioral data on the scanner task were not collected for 3 SZ participants and 2 HC participants due to technical error. SZ and HC did not differ in accuracy in either condition. SZ did not differ from HC in RT to the FP question, but were significantly slower to the FB question.

### 3.2 fMRI results

#### 3.2.1 Neural activity for FB > FP within each group

**Fig. 1:**
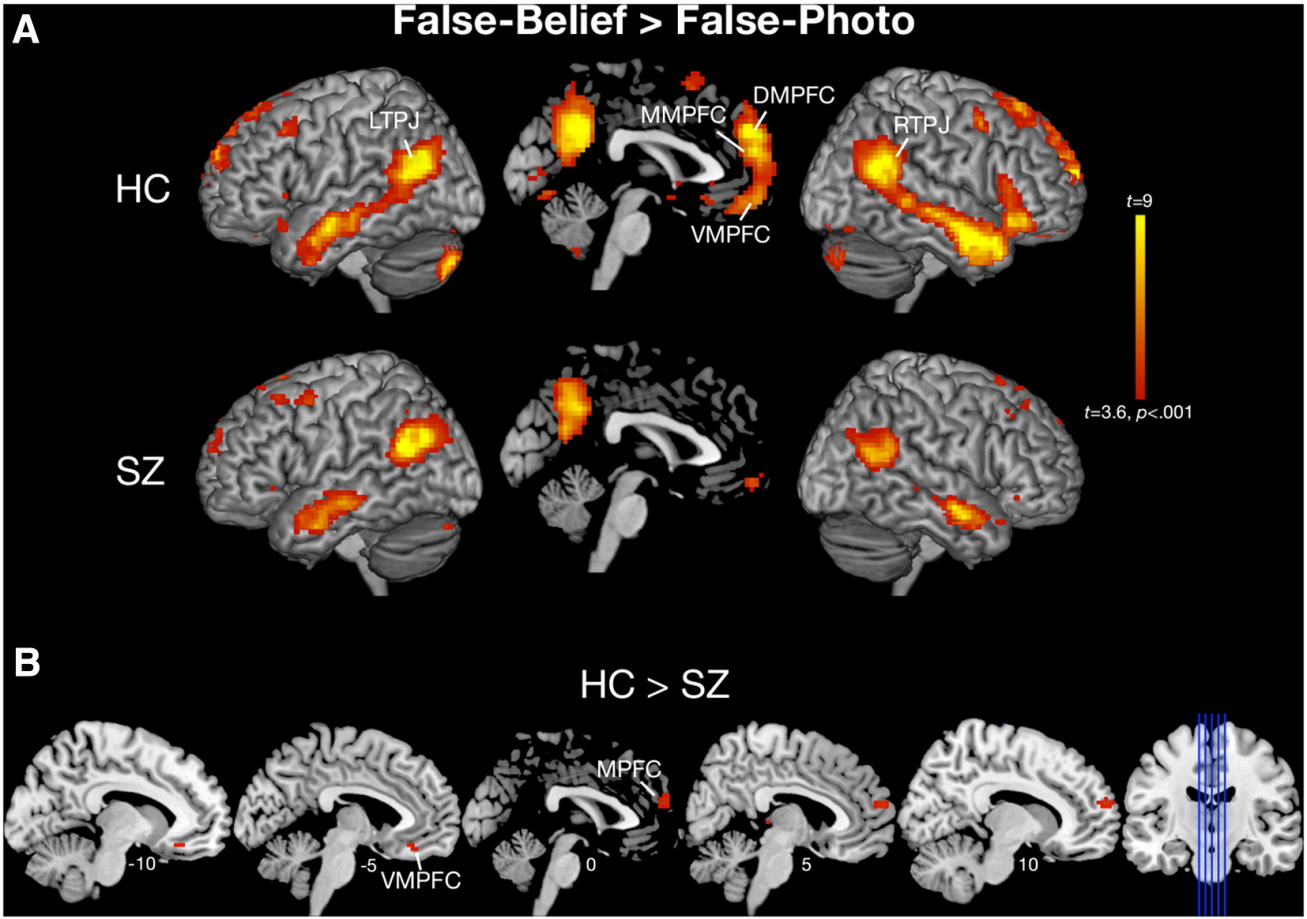
Whole-brain analysis. A) Top panel depicts within-group one-sample *t*-tests for False-Belief > False-Photograph. B) Bottom panel depicts full factorial ANOVA results demonstrating group ∗ condition interactions whereby controls showed greater activation for False-Belief > False-Photograph compared to individuals with schizophrenia. All images are displayed at *p* < .001, uncorrected for multiple comparisons.

Consistent with prior work using this task, in HC, contrasting activity for FB > FP revealed robust recruitment of the ToM network, including bilateral TPJ, and DMPFC extending to VMPFC (Table 2, Fig. 1A). A similar pattern of activation was observed in SZ, although less pronounced, particularly in MPFC.

#### 3.2.2 Between-group differences: ROI analysis

**Fig. 2:**
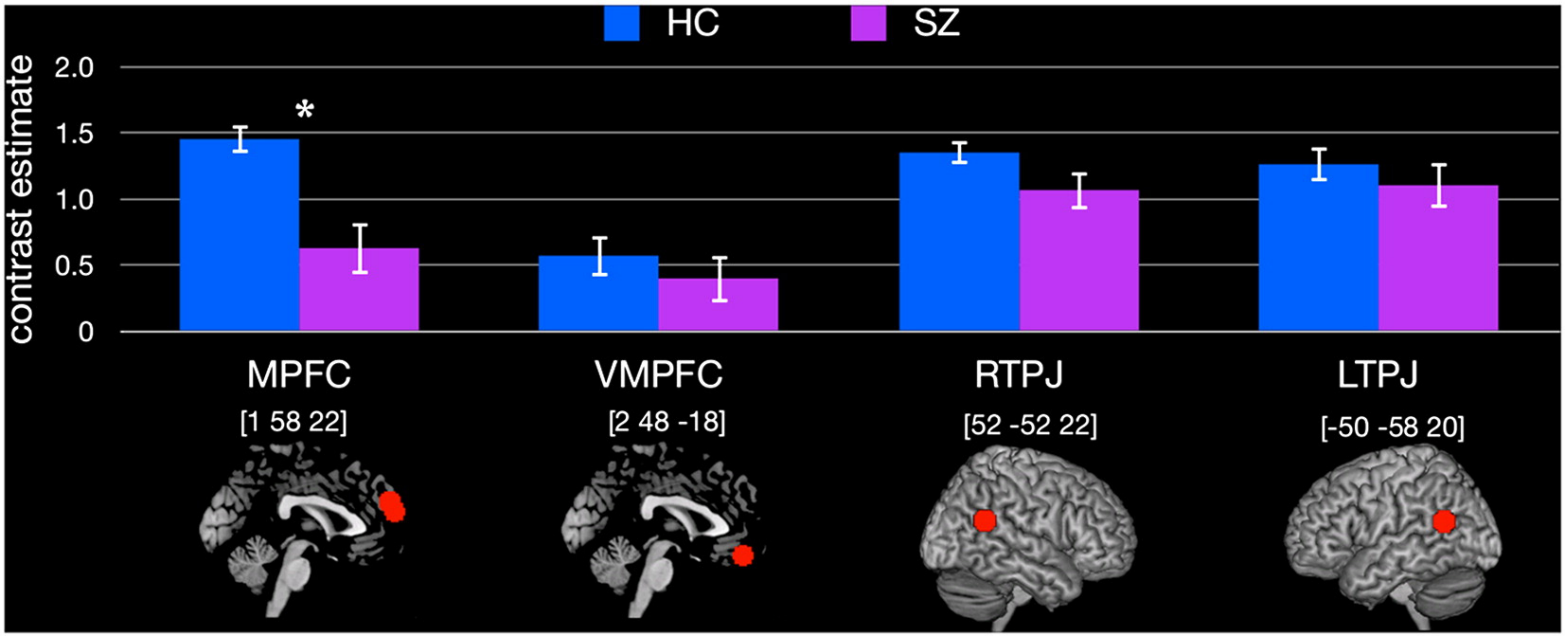
Region-of-interest analysis. Results from the independent region-of-interest (ROI) analysis in medial prefrontal cortex (MPFC), ventral medial prefrontal cortex (VMPFC), right temporo–parietal junction (RTPJ), and left temporo–parietal junction (LTPJ). The red spheres represent the regions from which contrast estimates were extracted. Montreal Neurological Institute (MNI) coordinates are displayed above each region. *p < .05.

Compared to HC, SZ exhibited reduced neural activity for FB > FP in MPFC, *t*(36) = 3.60, *p* = .001, *d* = 1.17, but not VMPFC, *t*(36) = .98, *p* = .33, *d* = .32, RTPJ, *t*(36) = 1.29, *p* = .20, *d* = .42 or LTPJ, *t*(36) = .90, *p* = .37, *d* = .29 (Fig. 2).

#### 3.2.3 Between-group differences: Whole-brain ANOVA

Exploratory analysis at *p* < .001, *k* > 10, uncorrected for multiple comparisons, revealed the predicted group ∗ condition interactions, whereby SZ exhibited reduced activity for FB > FP compared to HC, in MPFC and VMPFC/orbitofrontal cortex (Table 2, Fig. 1B). However, these differences did not survive correction for multiple comparisons. No regions showed the opposite interaction (i.e., SZ > HC for FB > FP).

### 3.3 Correlations between neural activity for ToM and social variables

We hypothesized that neural activity for ToM would be related to our measures of social behavior, including trait perspective-taking/empathy (IRI), social cognition (MSCEIT-ME), and social functioning (SAS, GFS). In line with this hypothesis, across all participants, neural activity for ToM in MPFC demonstrated significant relationships with IRI-PT, MSCEIT-ME, SAS, GFS such that greater neural activity for FB > FP was associated with better scores on these measures (Table 3). RTPJ activity correlated with MSCEIT-ME performance, SAS, and a trend level with IRI-PT. Partial correlations controlling for age and IQ did not change these findings except for the relationship between MPFC and IRI-PT, MPFC and GFS, and RTPJ and MSCEIT-ME, which were reduced to trend levels of significance (*p* < .10) (Supplementary Table 3). Correlations within SZ and HC are summarized in Table 3.

SocAnh was negatively associated with neural activity in MPFC such that greater SocAnh was associated with less neural activity for ToM. This association was not observed with VMPFC, RTPJ, or LTPJ activity.

### 3.4 Does neural activity for ToM mediate the relationship between social anhedonia and social functioning?

**Fig. 3:**
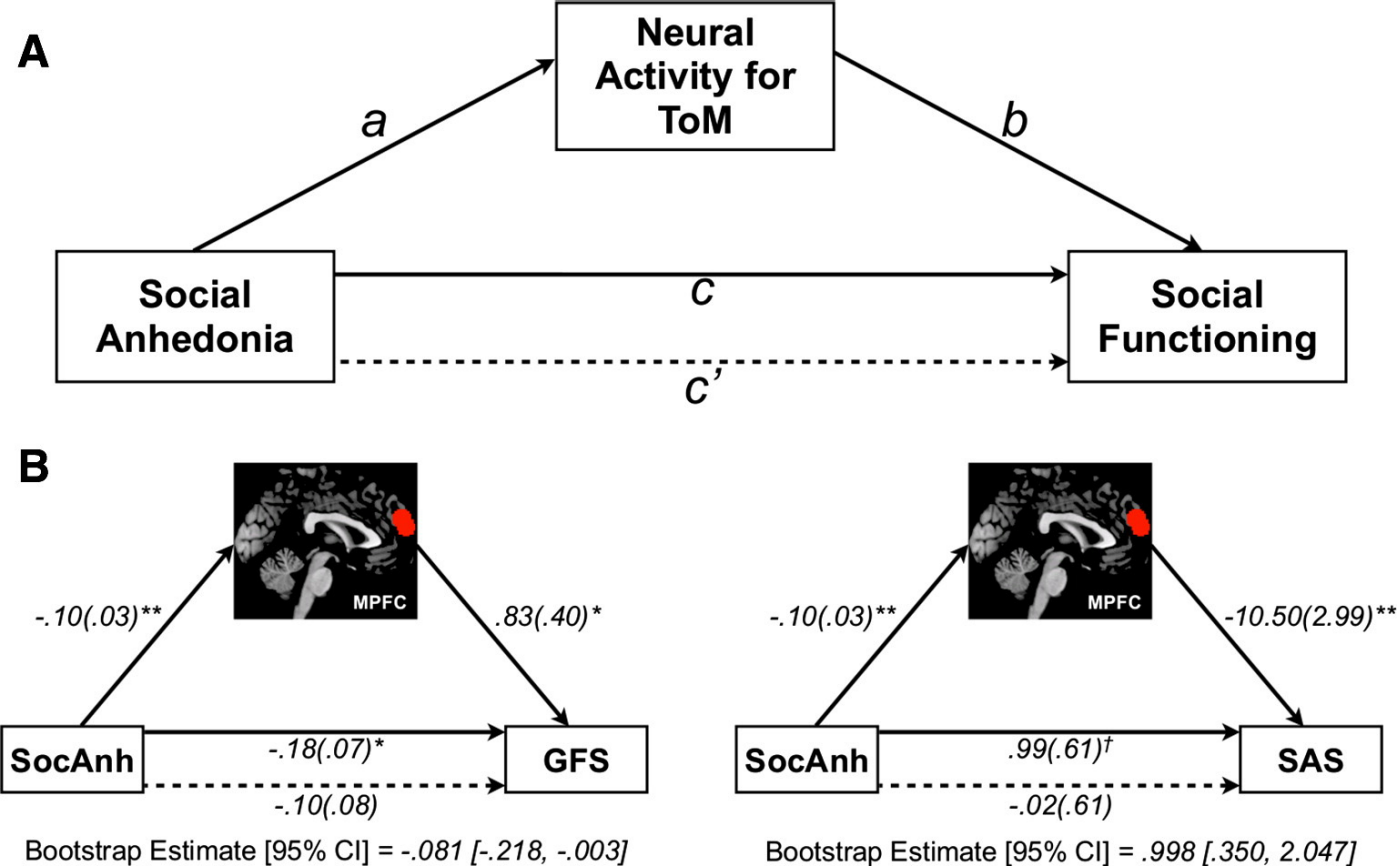
Mediation analysis. A) We tested whether neural activity for theory of mind (ToM) in medial prefrontal cortex (MPFC) mediated the relationship between social anhedonia (SocAnh) and our measures of social functioning (Social Adjustment Scale—Self-Report [SAS], Global Functioning Social Scale [GFS]). Path *c* is the *total effect* of social anhedonia on social functioning; path *c*′ is the *direct effect* of social anhedonia on social functioning after controlling for neural activity for ToM; the product of the paths *a* and *b* (*ab*) is the *indirect effect* of social anhedonia on social functioning, through neural activity for ToM. B) Results from the mediation effects of social anhedonia on GFS through MPFC activity (left), and mediation effects of social anhedonia on SAS through MPFC activity (right). Unstandardized path coefficients are displayed along with standard errors in parentheses. **p ≤ .01, *p ≤ .05, ^†^*p* = .11.

To address our second aim, we examined whether ToM-related activity mediates the relationship between social anhedonia and social functioning in those brain regions that demonstrated a significant relationship with both social anhedonia (path *a*) and social functioning (path *b*): MPFC (Fig. 3A). The addition of MPFC activity in the mediation model rendered the relationship between SocAnh and GFS (path *c*′) non-significant (Fig. 3B). Additionally, though the relationship between SocAnh and SAS was not statistically significant (*p* = .11), the addition of MPFC activity in the mediation model reduced the strength of this relationship. Bootstrap analysis of the *indirect effect* (path *ab*) revealed that MPFC activity accounted for a statistically significant portion of the variance in the relationship between SocAnh and GFS, and SocAnh and SAS.

## 4 Discussion

Using a well-validated ToM scanner task and multiple-methods for assessing different aspects of social behavior, we found that individuals with schizophrenia exhibit reduced recruitment of MPFC for ToM. Neural activity in MPFC correlated with understanding how to manage emotions (MSCEIT-ME), the tendency to engage in perspective-taking (IRI-PT), and both self-reported (SAS) and clinician-rated (GFS) measures of social functioning. Finally, mediation analysis provided evidence that social anhedonia influences social functioning through its effect on ToM-related neural circuitry.

Similar to other investigations (Brune et al., 2011, 2003; Das et al., 2012; Hooker et al., 2011; Lee et al., 2006, 2011; Walter et al., 2009a), the ROI analysis revealed significantly less MPFC activity for ToM in individuals with schizophrenia versus matched healthy controls. Though the whole-brain analysis did not yield any group differences at a corrected threshold, we observed largely converging evidence of reduced MPFC activity in the schizophrenia group at an uncorrected threshold. Importantly, we found several relationships between neural activity in MPFC and RTPJ for ToM and aspects of social behavior, which were largely unchanged when controlling for the effects of age and IQ. More specifically, neural activity in MPFC correlated with trait perspective-taking on the IRI-PT, MSCEIT-ME performance, self-reported social functioning on the SAS, and clinician-rated social functioning on the GFS, such that greater activity in these regions was associated with better social cognition and social functioning, respectively. RTPJ activity correlated with MSCEIT-ME, SAS, and IRI-PT at a trend level. We note that several of these relationships may have been influenced, in part, by group differences on the social variables (e.g., MSCEIT-ME, SAS, and GFS), which could have led to clustering of data points by group and an inflated correlation coefficient. Furthermore, most of these relationships were observed across all participants, and not separately within each group. With that said, we did find several significant associations within SZ participants alone: greater neural activity in MPFC was associated with less social impairment on the SAS, and greater neural activity in RTPJ was associated with better ability to manage emotions through the use of affective ToM skills on the MSCEIT-ME. The finding that some brain–behavior relationships were found in one group and not the other would suggest that there may be differences between the groups in how ToM-related neural activity may influence social behavior. However, reduced power may have prevented us from observing additional relationships between neural activity and social functioning within the SZ and HC group separately. Furthermore, recruitment of the ToM network should in theory be related to social functioning and ability regardless of diagnosis making it important to investigate these associations across groups where the relationship can be examined across the full range of neural and social functioning. Nonetheless, the relationships found here should be interpreted with caution, and examined in future work with larger sample sizes.

Together, these findings indicate that increased neural activity for ToM in MPFC and RTPJ is associated with greater perspective-taking in daily life, enhanced social cognitive ability, specifically the ability to use ToM skills to effectively manage emotions in different social situations, and social functioning. These findings are consistent with other studies that have found increased MPFC activity during a ToM task to predict improvements in social functioning following recovery from a psychotic episode (Lee et al., 2006), and, in a separate study, following cognitive remediation (Subramaniam et al., 2012). Studies have also shown that VMPFC gray matter volume correlates with engagement in ToM to enhance interpersonal relationships among individuals with schizophrenia (Hooker et al., 2011), and MPFC/TPJ activity during ToM tasks correlates with increased perspective-taking in daily life (Falk et al., 2012; Hooker et al., 2008; Masten et al., 2013; Moriguchi et al., 2006). These findings further highlight the functional significance of neural activity for ToM by relating it to several different aspects and measures of social behavior.

The relationship between MPFC and RTPJ activity and MSCEIT-ME performance is of particular significance. The MSCEIT-ME requires participants to use affective ToM (i.e., reason about the emotions of others) in order to effectively manage a story character's emotion and navigate various social situations. The correlations observed in the current study suggest that ToM-related neural circuitry may be important for social functioning by supporting affective ToM ability. These findings are of particular importance when considering that cognitive remediation programs, which include social cognition training, demonstrate intervention-related improvement on MSCEIT-ME performance (Eack et al., 2007, 2011; Hooker et al., 2012; Sacks et al., 2013) and increased MPFC activity which tracks with improvements in social functioning (Subramaniam et al., 2012). Taken with our data, it suggests that the neural mechanisms supporting ToM are amenable to intervention and are likely to result in measurable changes in affective ToM skills and the use of those skills to improve social interaction. Furthermore, in line with the MATRICS initiative (Green et al., 2004, 2005), these data, taken with other neuroimaging findings (Wojtalik et al., 2013), demonstrate that performance on, at least, the social cognition subtest of the MATRICS tracks with important individual differences in neurobiology that are linked to social functioning, supporting the measure's validity and usefulness as a tool to assess intervention-related change.

Addressing our second aim regarding the role of ToM-related neural circuitry in the social anhedonia—social functioning relationship, mediation analysis replicated findings demonstrating a link between social anhedonia and social functioning (Blanchard et al., 1998, 2011; Cohen et al., 2005), and provided novel evidence that 1) neural activity for ToM in MPFC is predicted by social anhedonia, such that individuals reporting higher social anhedonia had less MPFC activity for ToM, and 2) neural activity in this region accounts for the relationship between social anhedonia and social functioning. Specifically, we found neural activity in MPFC to account for a significant portion of the variance between social anhedonia and our measures of social functioning, including the SAS (self-report) and GFS (clinician-rated). This finding suggests that social anhedonia impacts social functioning, at least partially, through its effect on ToM-related neural circuitry.

The extant literature on social anhedonia and social functioning has largely focused on the role of decreased reward and impaired memory for reward (Dowd and Barch, 2010; Gard et al., 2007; Horan et al., 2008; Kring and Elis, 2012; Strauss and Gold, 2012). Here, we demonstrate a previously uninvestigated link between social anhedonia and social functioning through impaired ToM. Although the data here are cross-sectional and cannot speak to causality, nor do they fully account for social functioning impairments in schizophrenia, the results are consistent with several interpretations. Disruption to the neural mechanisms subserving ToM may make social interaction challenging, increase social stress, decrease social reward or increase disinterest in socializing, thereby contributing to anhedonia and social dysfunction. On the other hand, social anhedonia and isolation, both of which have been identified as risk factors for schizophrenia (Kwapil, 1998; Tarbox and Pogue-Geile, 2008; van Os et al., 2000), may lead to deleterious changes in the neural mechanisms subserving ToM, thereby contributing to misperceptions of intentions and emotions, interpersonal conflict, and social impairment (Hoffman, 2007). Though both explanations are theoretically viable, additional work, such as prospectively measured reports of social anhedonia, ToM-related neural function, and social functioning, would be needed in order to better evaluate these possibilities. Nonetheless, the findings do demonstrate significant interrelationships between these different constructs. Furthermore, it suggests that training ToM, as opposed to pharmacologically augmenting response in reward-related neural circuitry (Juckel et al., 2006a, 2006b; Schlagenhauf et al., 2008; Walter et al., 2009b; Waltz et al., 2007), may be a more accessible process for remediation that will most likely lead to improvements in social functioning.

It is important to note that functional outcome in schizophrenia is multiply determined. Neural function, while shown here to capture a significant portion of the variance in social functioning, still leaves much variance unexplained. Research has demonstrated that cognitive and affective factors such as dysfunctional attitudes (Granholm et al., 2009; Grant and Beck, 2009; Green et al., 2012; Horan et al., 2010), motivation (Gard et al., 2009), and metacognition (Lysaker et al., 2010a, 2010b, 2011), as well as external factors relating to social support (Brekke et al., 2005), disability policies and the availability of employment (Rinaldi et al., 2010; Tandberg et al., 2013), also contribute to functioning. A comprehensive model of functional outcome would ideally incorporate all of these factors. Similarly, functioning may best be improved through a multipronged approach targeting neurocognitive functioning as well as these other factors.

Finally, the sample of schizophrenia participants tested here demonstrated less neurocognitive impairment (i.e., IQ, MATRICS) than what is typically observed in this population (e.g., Kern et al., 2011). Though this may be in part the result of recruiting an education-matched HC group, it warrants caution in generalizing our findings to lower-functioning individuals with schizophrenia. With that said, the neural data demonstrating reduced recruitment of MPFC for ToM in the schizophrenia group replicates several other studies with more neurocognitively impaired schizophrenia samples. This suggests that reduced MPFC activity for ToM is characteristic of individuals with schizophrenia high- or low-functioning.

In conclusion, the current study finds that individuals with schizophrenia exhibit reduced recruitment of MPFC for ToM, which is related to social functioning, and may be a contributing mechanism through which social anhedonia affects social functioning. These findings reveal proximal neurobiological determinants of social functioning, indicative of aberrant function of the social brain, and suggest neural targets for remediation.

## Figures and Tables

**Table 1:** Participant characteristics, social variables, and False-Belief Task performance.

	SZ	HC	Between-group difference
*n*	20	18	
Gender (male/female)	12/8	12/6	χ^2^ (1, N = 38) = .181, *p* = .671
Age	38.8 (9.7)	32.4 (12.1)	*t*(36) = 1.78, *p* = .084
Education (years)	15.0 (2.3)	14.2 (2.6)	*t*(36) = 1.00, *p* = .326
IQ^a^	108.7 (13.4) [86–133]	107.4 (10.7) [89–123]	*t*(36) = 0.30, *p* = .763, *d* = .10
SocAnh	8.2 (3.4) [3–19]	5.9 (4.3) [1–18]	*t*(36) = 1.79, *p* = .082, *d* = .58
Diagnosis (*n*)			
Schizophrenia	16 (80%)		
Schizoaffective	4 (20%)		
Duration of illness (years)^b^	17.1 (12.2)		
CPZ equivalent^c^	501.6 (402.8)		
PANSS symptoms			
Positive symptoms	3.1 (1.1)		
Negative symptoms	1.7 (0.6)		
Disorganized symptoms	1.5 (0.8)		
Neurocognition—MATRICS^d^			
Speed of processing	46.7 (9.7)	54.3 (8.1)	*t*(36) = 2.62, *p* = .013, *d* = .85
Attention/vigilance	45.5 (10.0)	49.7 (9.2)	*t*(36) = 1.35, *p* = .185, *d* = .44
Working memory	51.2 (8.2)	49.4 (8.9)	*t*(36) = .63, *p* = .530, *d* = .21
Verbal learning	46.8 (10.2)	52.9 (9.7)	*t*(36) = 1.90, *p* = .066, *d* = .62
Visual learning	44.0 (11.2)	47.8 (10.1)	*t*(36) = 1.12, *p* = .270, *d* = .36
Reasoning and problem solving	46.4 (10.2)	50.2 (7.9)	*t*(36) = 1.30, *p* = .204, *d* = .42
Social variables			
IRI-PT	30.3 (4.4) [20–37]	32.7 (4.6) [24–42]	*t*(36) = 1.658, *p* = .106, *d* = .54
IRI-EC	31.1 (5.5) [21–42]	31.9 (4.8) [24–42]	*t*(36) = 0.50, *p* = .623, *d* = .16
MSCEIT-ME (*T* score)	38.9 (8.6) [22–54]	55.6 (9.6) [34–70]	*t*(36) = 5.67, *p* < .001, *d* = 1.83
SAS (*T* score)	66.4 (17.1) [43–97]	53.8 (9.4) [40–76]	*t*(36) = 2.78, *p* = .009, *d* = .92
GFS	6.2 (1.8) [3–9]	8.3 (1.4) [6–10]	*t*(36) = 4.05, *p* < .001, *d* = 1.33
False-Belief Task^e^			
FB accuracy (%)	74.8 (17.3)	80.6 (16.0)	*t*(31) = 0.99, *p* = .330, *d* = .34
FP accuracy (%)	79.9 (15.3)	82.2 (14.3)	*t*(31) = 0.45, *p* = .656, *d* = .16
FB RT (s)	4.1 (0.6)	3.4 (0.6)	*t*(31) = 3.36, *p* = .002, *d* = 1.17
FP RT (s)	3.7 (0.5)	3.4 (0.5)	*t*(31) = 1.30, *p* = .202, *d* = .45

*Note*. Unless otherwise indicated, values represent means with standard deviations in parentheses and the range in square brackets. SZ = schizophrenia, HC = healthy control, SocAnh = Revised Social Anhedonia Scale, CPZ = chlorpromazine, PANSS = Positive and Negative Symptom Scale, IRI-PT = Interpersonal Reactivity Index—Perspective Taking, IRI-EC = Interpersonal Reactivity Index—Empathic Concern, SAS = Social Adjustment Scale—Self-Report, GFS = Global Functioning Social Scale, MSCEIT-ME = MSCEIT—Managing Emotions subtest of the MATRICS, FB = False-Belief, FP = False-Photograph.

a IQ was estimated from the vocabulary and matrix reasoning subtests of the WASI.

b Data were not collected from 1 participant.

c Two patients were not taking medication.

d *T* scores.

e Due to technical error, data were not collected for 3 SZ and 2 HC participants.

**Table 2:** Results from the whole-brain analyses comparing neural activity for FB > FP within and between groups.

Region	BA	Volume in voxels	MNI coordinates *x y z*	*T* value
*HC*				
R precuneus⁎		782	6 − 55 34	14.33
R anterior superior temporal sulcus⁎	20	1616	51 − 1 − 26	13.87
R posterior superior temporal sulcus⁎	21	–	51 − 25 − 8	12.12
R temporo–parietal junction⁎	21	–	54 − 58 19	10.79
Dorsal medial prefrontal cortex⁎		1293	0 53 31	13.84
R middle medial prefrontal cortex⁎	10	–	9 65 16	10.89
R ventral medial prefrontal cortex⁎	11	–	3 56 − 14	7.93
L temporo–parietal junction⁎	39	960	− 48 − 64 22	11.61
L cerebellum⁎		220	− 24 − 76 − 38	10.20
L cerebellum⁎		39	− 6 − 58 − 44	6.60
R middle frontal gyrus⁎	6	39	45 8 49	6.48
R cerebellum⁎		134	21 − 76 − 29	6.48
R lingual gyrus⁎	17	69	6 − 76 − 11	6.43
L middle frontal gyrus⁎	46	36	− 36 20 43	6.23
L middle frontal gyrus	9	21	− 21 26 43	5.54
L inferior frontal gyrus	48	15	− 30 17 − 17	5.10
L thalamus		10	− 9 − 13 10	4.97
R rostral anterior cingulate cortex	11	15	3 26 − 11	4.89

*SZ*				
L temporo–parietal junction⁎	39	537	− 51 − 55 25	12.17
L precuneus⁎		697	− 3 − 58 40	8.88
R anterior superior temporal sulcus⁎	21	176	60 − 7 − 14	8.79
R temporo–parietal junction⁎	22	410	51 − 49 19	8.29
L anterior superior temporal sulcus⁎	21	295	− 54 − 1 − 26	7.25
L superior frontal gyrus	8	24	− 6 20 64	6.01
L ventral medial prefrontal cortex	11	26	− 3 56 − 11	5.73
R middle medial prefrontal cortex	10	34	9 53 10	5.21
L middle frontal gyrus	6	24	− 39 2 52	5.10
L cerebellum		36	− 24 − 76 − 32	4.96
R posterior superior temporal sulcus	21	17	54 − 34 − 2	4.70
L dorsal medial prefrontal cortex⁎	10	52	− 6 59 25	4.61
L middle frontal gyrus	46	13	− 33 23 55	4.52
R middle frontal gyrus	9	22	27 23 43	4.50
R superior frontal gyrus	9	16	21 35 43	4.00

*HC > SZ*				
R medial prefrontal cortex	10	44	9 62 19	4.26
Medial prefrontal cortex	10	–	0 56 19	4.15
L ventral medial prefrontal cortex	11	10	− 9 35 − 14	3.84

*Note*. Statistical threshold is *p* < .001, *k* = 10/270 mm, uncorrected for multiple comparisons. Dash (–) in the volume column indicates that the region is included in the cluster above. BA = Broadmann area, MNI = Montreal Neurological Institute, HC = healthy control group, SZ = schizophrenia group, R = right, L = left.

⁎ Asterisks next to the anatomical region indicates that the region is significant at a voxel-wise threshold of *p* < .001 corrected at the cluster-level to *p* < .05.

**Table 3:** Correlations between neural activity in the ToM ROIs and the social variables.

	MPFC			VMPFC			RTPJ			LTPJ		
	All	HC	SZ	All	HC	SZ	All	HC	SZ	All	HC	SZ
IRI-PT	.33⁎	.68⁎⁎	− .03	.20	.50⁎	.00	.28†	.36	.16	.13	.19	.03
IRI-EC	.24	.42†	.14	.21	.27	.18	− .21	− .22	− .24	− .22	− .22	− .24
MSCEIT-ME	.53⁎⁎	.33	.28	.04	.32	− .36	.33⁎	− .13	.54⁎	.16	− .02	.16
SAS	− .56⁎⁎	− .43†	− .44⁎	− .11	− .39	.05	− .33⁎	− .39	− .23	− .21	− .07	− .21
GFS	.45⁎⁎	.47⁎	.11	.02	.19	− .19	.15	− .07	.09	.07	− .10	.04
SocAnh	− .48⁎⁎	− .55⁎	− .33	− .27	− .41†	− .09	− .10	.18	− .23	.19	.13	.37

*Note*. Pearson *r* values depicted. MPFC = medial prefrontal cortex, VMPFC = ventral medial prefrontal cortex, RTPJ = right temporo–parietal junction, LTPJ = left temporo–parietal junction, All = all participants, HC = healthy control group, SZ = schizophrenia group, IRI-PT = Interpersonal Reactivity Index—Perspective Taking, IRI-EC = Interpersonal Reactivity Index—Empathic Concern, MSCEIT-ME = Managing Emotions branch of the MSCEIT, SAS = Social Adjustment Scale—Self-Report, GFS = Global Functioning Social Scale, SocAnh = Social Anhedonia.

⁎⁎ *p* < .01.

⁎ *p* < .05.

† *p* < .10.
