# Supplementary material for: The neural basis of theory of mind and its relationship to social functioning and social anhedonia in individuals with schizophrenia
Source: Neuroimage Clin. 2013 Nov 27;4:154–63. doi: 10.1016/j.nicl.2013.11.006 (PMC3871293; doi:10.1016/j.nicl.2013.11.006)
Supplement: Supplementary file 1 — Supplementary tables. [file mmc1.docx]

**Supplementary Table 1**

*Brain regions demonstrating greater activity in HC versus SZ for FB>FP, controlling for age*

| Region | BA | Volume in Voxels | MNI coordinates  *x y z* | *T* Value |
| --- | --- | --- | --- | --- |
| R Medial Prefrontal Cortex | 10 | 46 | 9 65 19 | 4.28 |
| Medial Prefrontal Cortex | 10 | - | 0 56 19 | 4.20 |
| L Ventral Medial Prefrontal Cortex | 11 | 10 | -9 35 -14 | 3.85 |

*Note*. Analysis was performed with whole-brain ANCOVA. Statistical threshold is *p*<.001, *k*=10/270mm, uncorrected for multiple comparisons. Dash (-) in the volume column indicates that the region is included in the cluster above. BA = broadmann area, MNI = Montreal Neurological Institute, HC = healthy control group, SZ = schizophrenia group, R = right, L = left.

**Supplementary Table 2**

*ROI analysis controlling for age*

| **Region** | **Between-Group Difference** |
| --- | --- |
| MPFC | *F*(1, 35)=8.79, *p*=.005, η^2^_p_=.20 |
| VMPFC | *F*(1, 35)=0.71, *p*=.406, η^2^_p_=.02 |
| RTPJ | *F*(1, 35)=1.11, *p*=.299, η^2^_p_=.03 |
| LTPJ | *F*(1, 35)=0.71, *p*=.405, η^2^_p_=.02 |

*Note*. Analysis was performed with ANCOVAs. MPFC = medial prefrontal cortex, VMPFC = ventral medial prefrontal cortex, RTPJ = right temporo-parietal junction, LTPJ = left temporo-parietal junction.

**Supplementary Table 3**

*Partial correlations controlling for age and IQ between neural activity in the ToM ROIs and the social variables*

|  | MPFC | | | VMPFC | | | RTPJ | | | LTPJ | | |
| --- | --- | --- | --- | --- | --- | --- | --- | --- | --- | --- | --- | --- |
|  | All | HC | SZ | All | HC | SZ | All | HC | SZ | All | HC | SZ |
| IRI-PT | .32^†^ | .65** | .14 | .18 | .43^†^ | -.09 | .26 | .37 | .21 | .11 | .22 | .02 |
| IRI-EC | .27 | .36 | .39 | .21 | .19 | .18 | -.21 | -.25 | -.20 | -.22 | -.25 | -.21 |
| MSCEIT-ME | .39* | .03 | .16 | .11 | .21 | -.30 | .31^†^ | -.19 | .58* | .18 | .05 | .21 |
| SAS | -.56** | -.50* | -.34 | -.14 | -.41 | -.10 | -.35* | -.40 | -.27 | -.23^†^ | -.11 | -.33 |
| GFS | .32* | .24 | -.03 | .03 | .01 | -.10 | .13 | -.13 | .12 | .13 | -.05 | .15 |

*Note*. MPFC = medial prefrontal cortex, VMPFC = ventral medial prefrontal cortex, RTPJ = right temporo-parietal junction, LTPJ = left temporo-parietal junction, All = All participants, HC = healthy control group, SZ = schizophrenia group, IRI-PT = Interpersonal Reactivity Index – Perspective Taking, IRI-EC = Interpersonal Reactivity Index – Empathic Concern, MSCEIT-ME = Managing Emotions branch of the MSCEIT, SAS = Social Adjustment Scale – Self-Report, GFS = Global Functioning Social Scale.

*** p*<.01

* *p*<.05

^†^ *p*<.10
